# Deep Vein Thrombosis (DVT) Prophylactic Team Activity to Support DVT Prevention Protocol for the Purpose of the Prophylaxis of Pulmonary Thromboembolism (PTE) and Operation

**DOI:** 10.3400/avd.oa.21-00017

**Published:** 2021-06-25

**Authors:** Shozo Tamura, Mai Yamamoto, Atsushi Kitagawa, Toshihiko Nagao

**Affiliations:** 1 Department of Medical Safety room, Ako Central Hospital, Ako, Hyogo, Japan; 2 Department of Nursing, Ako Central Hospital, Ako, Hyogo, Japan; 3 Department of Cardiovascular Surgery, Ako Central Hospital, Ako, Hyogo, Japan

**Keywords:** deep vein thrombosis, pulmonary thromboembolism, protocol, Wells’ score for DVT, prophylactic team

## Abstract

**Objective:** In 2017, the Medical Accident Investigation and Support center in Japan released an analysis of acute pulmonary thromboembolism (PTE) related mortality. This recommendation called for maintaining a “team in charge of PTE’s risk assessment, prevention, diagnosis and treatment” and preventing PTE through team activities. Therefore, we recommended establishing a deep vein thrombosis (DVT) prevention team. Before this recommendation, a multidisciplinary DVT prevention team was established in our hospital, with excellent outcomes. In the current study, we report the results of the DVT prevention team.

**Methods:** Our multidisciplinary team consisted of several departments: Cardiovascular Surgery, ward nurses, medical safety managers, and clerks. The following themes were launched: 1) preparation of DVT prevention protocol; 2) preparation of DVT preventive manual; 3) regular round for evaluating DVT preventive measures; 4) staff education. The protocol’s strong point was that nurses evaluated patients over 16-year-old with Wells’ score for DVT on admission. We retrospectively investigated the diagnosis rate of DVT and PTE for 9 months before and after protocol operation.

**Results:** The diagnosis rate of DVT was significantly improved after protocol implementation (before: 0.06% vs. after: 0.56%, p=0.0017). However, no significant difference was observed in the diagnosis rate of PTE before and after the protocol execution (before: 0.03% vs. after: 0.07%, p=0.98).

**Conclusion:** Our DVT prophylactic protocol improved the diagnostic rate of DVT resulting in a decrease of PTE in our hospital. (This is a translation of Jpn J Phlebol 2019; 30(3): 285–293.)

## Introduction

In August 2017, Japan Medical Safety Research Organization published a recommendation to prevent recurrence of medical accidents: “Analysis of Deaths Related to Acute Pulmonary Embolism.”^1)^ According to this recommendation, the number of pulmonary embolism (PTE) cases in Japan has increased 4.6 times in the past 15 years,^2)^ and the mortality rate of acute pulmonary embolism is 60% once it occurs falls into cardiac arrest.^3)^ This indicates the importance of preventive measures for deep vein thrombosis (DVT) in the lower limbs and pelvis, which are embolus sources. Also, “understanding of risks and recognition of diseases,” “prevention,” “early detection/early diagnosis,” “initial treatment,” and “improvement of the in-hospital system” are necessary to prevent accident recurrence. It is essential to avoid death from the onset of acute pulmonary embolism from the hospital. Before these recommendations, the hospital established a project team (“DVT Prevention Team”), under the Medical Safety Management Office, to prevent DVT, the root cause of acute pulmonary embolism.

We report a retrospective study of the change in the number of DVT and PTE diagnoses to determine the outcomes of our original DVT prevention protocol and the multifaceted interventions of our DVT prevention team, which were designed and implemented based on Japanese guidelines and field conditions.^4,5)^

## Materials and Methods

### Materials

**Figure 1:**
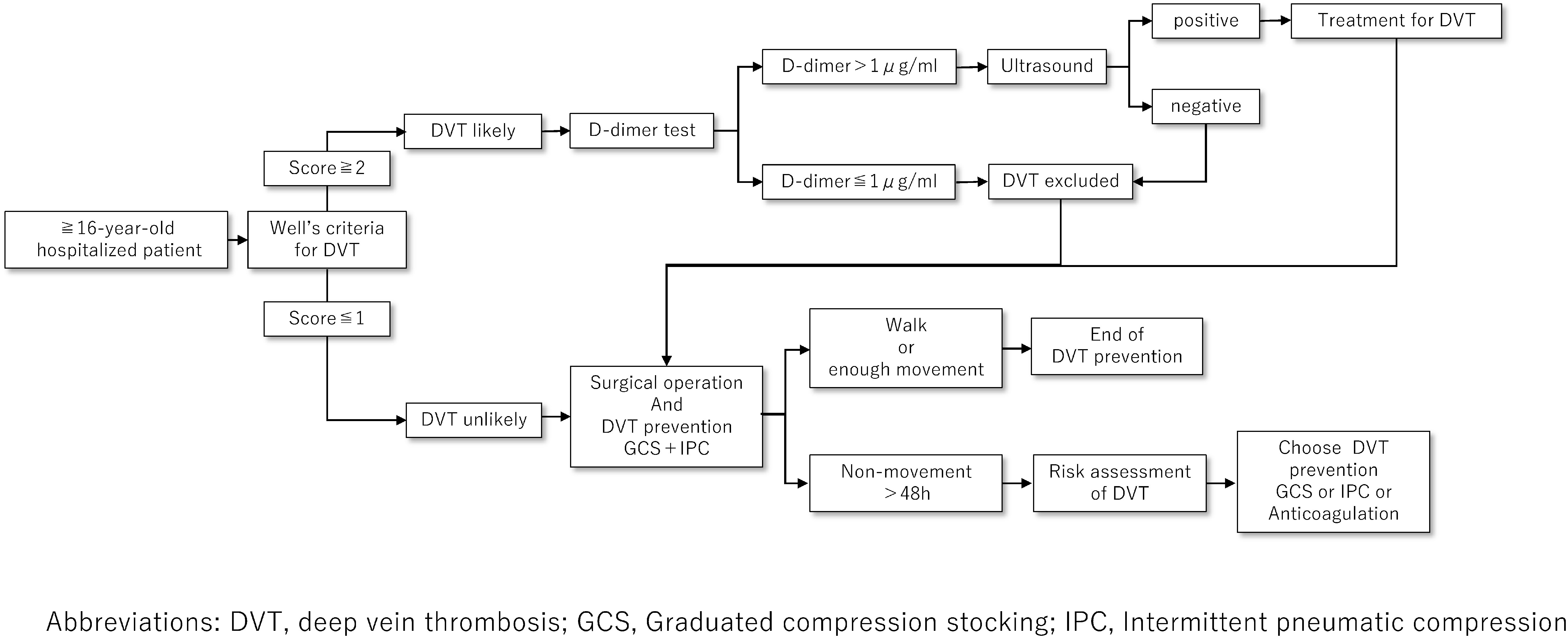
Fig. 1 Flowchart representing the surgical protocol of DVT.

**Figure 2:**
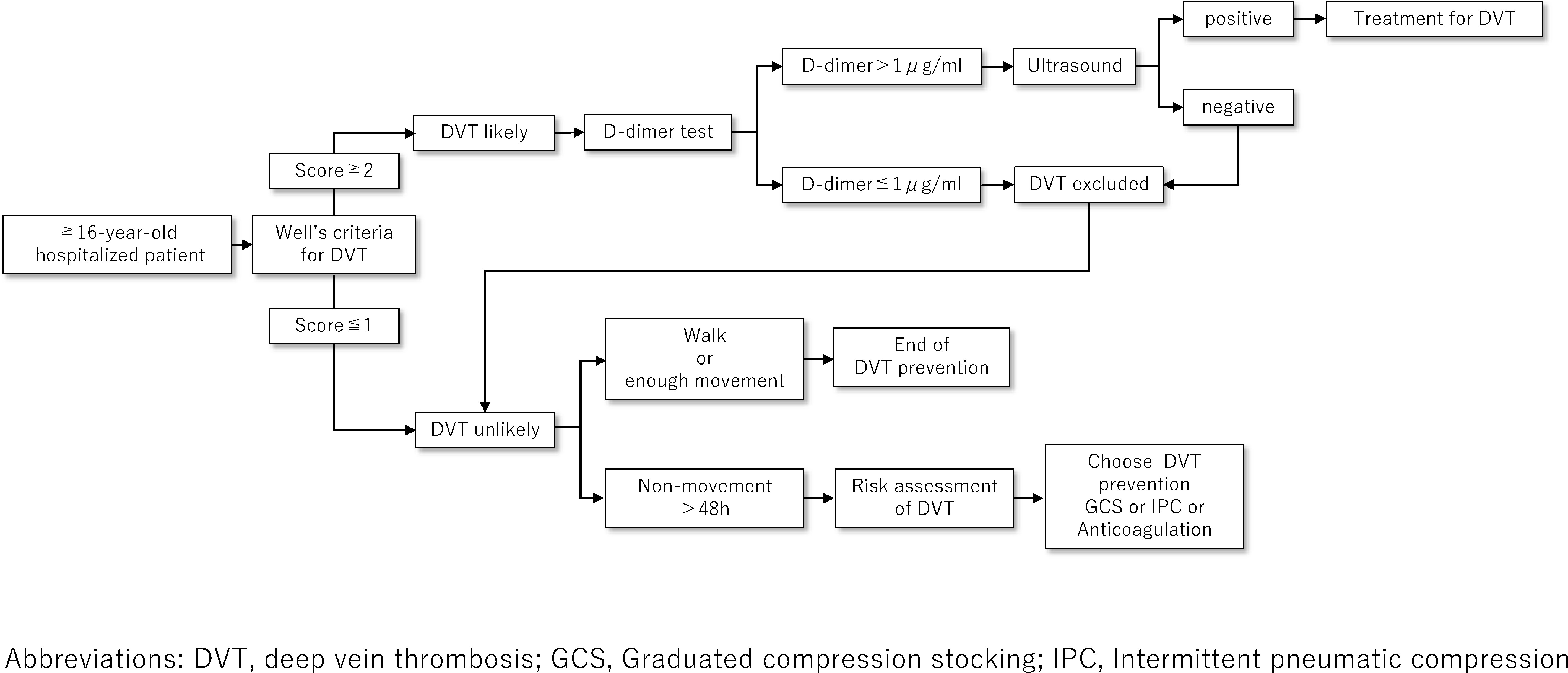
Fig. 2 Flowchart representative of the non-surgical protocol of DVT.

Measures were implemented for 2,992 inpatients aged 16 years and older at our hospital between April 2018 and December 2018, according to the surgical (**Fig. 1**) and non-surgical (**Fig. 2**) cases of DVT prevention protocol we developed. The hospital Ethics Committee approved this study (IRB: 20181124).

### Methods

Before developing the DVT prevention protocol, the hospital appointed a DVT prevention team (consisting of a cardiologist, 7 nurses from each ward, a clerk, and a clinical engineer) to implement DVT prevention activities for the entire organization. The main activities are (1) development of DVT prevention protocol, (2) development of DVT prevention manual, (3) ward rounds, and (4) educational activities for staff.

### DVT prevention protocol

The DVT Prevention protocols (surgical and non-surgical) made by our DVT prevention team are shown (surgical, **Fig. 1** and non-surgical, **Fig. 2**). The patients’ target for risk assessment and prevention of DVT are adults (age>16 years old) regardless of daily activities. To the patients, screening and diagnostic test for DVT, assessment of the risk of DVT, and prevention of DVT based on the patients’ risk, according to the DVT prevention surgical or non-surgical protocol.

### The surgical protocol of DVT

As a primary screening, on the day the patient is admitted, the nurse in charge of the inpatient ward assesses the clinical potential of DVT using the criteria: Wells’ score for DVT (**Table 1**). Suppose the patient has the Wells’ score of ≤1 on the first day of hospitalization. In that case, the clinical probability of DVT is considered low, and the patient is fitted with elastic stockings preoperatively. An intermittent pneumatic compression device is applied in the operating room, which is continued until the patient can walk postoperatively. Continue wearing until the device is worn, and you can walk after surgery. If the primary screening indicates a high clinical probability of DVT (Wells’ score ≥2 points), first report to the attending physician. D-dimer and lower extremity venous ultrasonography (D-dimer >1 µg/mL confirms the diagnosis by lower extremity venous ultrasonography; D-dimer ≤1 µg/mL denies the presence of DVT) for the diagnosis of DVT.

**Table 1:** Table 1 Wells’ score for DVT

Clinical Characteristics	Score
Active cancer (patient received treatment for cancer within the previous 6 months or is currently receiving palliative treatment)	1
Paralysis, paresis, or recent plaster immobilization of the lower extremities	1
Recently bedridden for 3 days or more, or major surgery within the previous 12 weeks requiring general or regional anesthesia	1
Localized tenderness along the distribution of the deep venous system	1
Leg swelling	1
Calf swelling at least 3 cm larger than that on the asymptomatic side (measured 10 cm below tibial tuberosity)	1
Pitting edema confined to the symptomatic leg	1
Collateral superficial veins (non varicose)	1
Previously documented deep-vein thrombosis	1
Alternative diagnosis to DVT is at least as likely	−2

The CT angiography from the pulmonary artery to the whole venous system of the lower body (inferior vena cava to iliac/femoropopliteal vein) is added to assess the PTE and/or DVT if necessary. A patient diagnosed with DVT undergoes DVT treatment: anticoagulant therapy, compression therapy, and placement of inferior vena cava filter, based on his or her disease severity (e.g., the thrombus is located central or peripheral vein, or the DVT phase is acute or chronic.). After that, the planned surgery will be scheduled.

Even with the Wells’ score of 0 to 1 on admission, the thrombus formation risk increases during the preoperative waiting period if the patient remains in bed for more than 48 h postoperatively or if the patient’s condition will require prolonged bedrest, such as a femoral fracture.

At this point, the nurse assesses the patient’s specific risk of developing DVT (**Table 2**) based on the Venous Thromboembolism Risk Assessment Table proposed by Kobayashi^7)^ The assessment results are reported to the attending physician, who directs DVT prevention measures (**Table 3**). The evaluation method is proposed by Kobayashi. Uses different evaluation tables for surgical and non-surgical cases to derive patient-specific risk of developing DVT. However, our risk evaluation method prevents confusion at the time of introduction. Therefore, priority is given to simplicity, and it is used in the evaluation table for non-surgical cases. For example, according to the evaluation method proposed by Kobayashi, the risk of developing DVT varies depending on the surgical procedure and surgical site in surgical cases. Under this hospital’s risk evaluation method, the risk of developing DVT varies mostly depends on the underlying disease. Since the operation is based on non-surgical cases, the risk does not change depending on the surgical procedure and surgical site, even in surgical cases.

**Table 2:** Table 2 Risk assessment scale for DVT

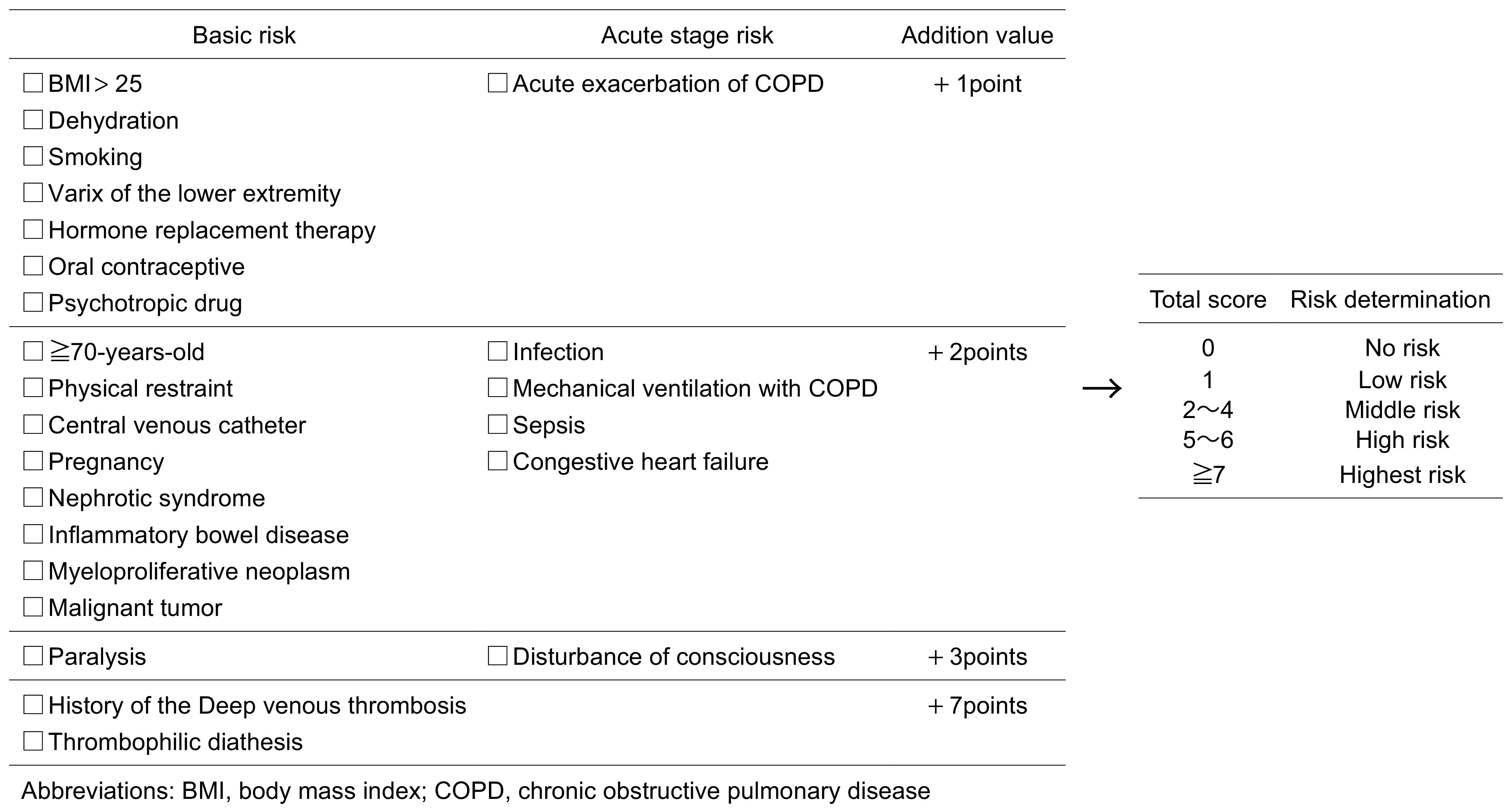

**Table 3:** Table 3 Methods of prophylaxis against DVT risk

DVT risk	Methods of prophylaxis against DVT risk
Low	Early exit from bed and enough movement
Middle	Graduated compression stockings (GCS) or Intermittent pneumatic compression (IPC)
High	GCS and IPC
Highest	GCS, IPC and Anticoagulation

Abbreviations: DVT, deep vein thrombosis; PTE, pulmonary thromboembolism; GCS, Graduated compression stocking; IPC, Intermittent pneumatic compression

### The non-surgical protocol of DVT

Patients eligible for evaluation and prophylaxis are the same as in surgical cases. On the day of admission, the patient is screened using the Wells’ score, and if DVT is present, appropriate treatment, such as anticoagulation, is administered as in surgical cases. If the patient does not have DVT, the nurse will assess the patient’s specific risk of developing DVT when the patient is expected to remain in bed for more than 48 h or to stay in bed for a long time. The results will be reported to the attending physician, who plans DVT prevention measures.

### DVT prevention manual

The DVT prevention manual is composed of the following: the explanation of the surgical or non-surgical protocol of DVT prevention, the method of assessment and cations related to Wells’ score for DVT, the method of assessment and cautions of DVT risk of the patients, the indication/ prohibits/ steps of DVT prevention based on the patient’s risk, and the works of doctor/ nurse in terms of DVT prevention (the explanation of the details of e-care records, etc.). The Wells’ score and the assessment results of the patient’s DVT risk are electronically recorded with e-care templates, which can be shared among various types of hospital staff.

### The ward rounds

The ward rounds are conducted to pick up the problems related to patient care and to pay attention to DVT prevention. Every month, we round each ward and see the patients at the bedside before a monthly conference to check the DVT prevention program’s state of achievement and validity. The DVT prevention team members collect the information about DVT prevention in advance and are ready to make a presentation on the ward round. We directly advise the attending nurse and try to improve the quality of care related to DVT prevention.

### Educational activities to the hospital staff

We conducted educational activities for the doctors, nurses, and physical therapists three times prior to the introduction of the DVT prevention system into our hospital. (The themes were the management of the DVT prevention protocol, the method of assessment of Wells’ score, and the right way of weaning compression stockings). We attend and do educational activities about DVT prevention for every doctor in our hospital conference. After introducing the DVT prevention protocol to our hospital, we willingly do educational activities, such as introducing the DVT prevention protocol into the new nursing staff’s educational program. Also, we irregularly publish the intrahospital newsletters related to the DVT occurrence rate and our hospital’s DVT prevention tasks and measures.

### Study design and patients

The observation period was 9 months before and after implementing the DVT prevention protocol (preoperative group: July 01, 2016, to March 31, 2017; postoperative group: April 01, 2018, to December 31, 2018; 274 days in both groups). The number of DVT diagnoses and diagnosis rates (number of diagnoses (cases)/number of hospitalized patients aged 16 years and older (persons)) the number of PTE diagnosis and diagnosis rates, and DVT diagnosis rates by lower extremity venous ultrasonography (number of diagnoses (cases)/number of lower extremity venous ultrasonography performed (cases)) were retrospectively investigated and compared. Also, the backgrounds of the patient groups before and after the operation (age, gender, surgery/non-surgery, cancer, and department) and the demographic characteristics (age, gender, surgical/non-surgical, admission from the retirement home, department, cancer, Wells’ score for DVT, type of DVT, clinical sign of DVT, D-dimer (µg/mL), risk determination of DVT, methods pf prophylaxis DVT, date of diagnosis with DVT (after hospitalization), with PTE, clinical sign of PTE). The Wells’ score was administered to eligible patients after operation, but we did not investigate the number of cases by score in this study. The number of DVT and PTE diagnoses before and after implementing the protocol was analyzed using EZR^6)^ (Saitama Medical Center, Jichi Medical University, Japan) with Pearson’s chi-square test, and p<0.05 was considered to be a statistically significant difference. The D-dimer test was performed using a Sysmex Rias Auto D-dimer neo, and was analyzed by the Line immunoassay method: LIA. The presence or absence of malignant neoplasms in patients’ comparison group was determined using the International Classification of Disease: ICD-10.

## Results

### Patient background

**Table 4** shows patient backgrounds for comparison. The number of patients aged 16 years or older was 2,894 in the preoperative group and 2,992 in the postoperative group during the period. The average age of the preoperative age group was 64.0±20.6, male/female ratio (44.2% male/55.8% female), surgical/non-surgical cases (37.7% surgical/62.3% non-surgical), cancer (7.2% of patients with previous history, 92.8% of patients without previous history), the department was internal medicine (34.0%), gynecology (19.2%), surgery (14.5%), orthopedics (9.6%). The average age of the postoperative age group was 62.7±21.3, male/female ratio (45.1% male/54.9% female), surgical/non-surgical cases (36.2% surgical/63.8% non-surgical), cancer (10.2% of patients with previous history, 89.8% of patients without previous history), the department was internal medicine (33.3%), gynecology (21.5%), surgery (12.4%), orthopedics (9.4%). Statistically significant differences included a higher mean age (64.0±20.6 vs. 62.7±21.3, p=0.017), significantly more surgical patients (420 (14.5%) vs. 370 (12.4%), p=0.018), and history of malignant neoplasms (209 patients (7.2%) vs. 304 patients (10.2%), p<0.001), and significantly more obstetrics and gynecology patients (555 patients (19.2%) vs. 644 patients (21.5%), p=0.028) in the preoperative group.

**Table 4:** Table 4 The demographic characteristics of patients before/after execution of DVT prevention protocol. Values are expressed as mean±SD or numbers and percentage (in brackets).

Patient Demographics	Before protocol (n=2894)	After protocol (n=2992)	P value
Age			
average±SD	64.0±20.6	62.7±21.3	0.017*
Gender			
Male	1278 (44.2)	1348 (45.1)	0.507
Female	1616 (55.8)	1644 (54.9)	
Surgical/non-surgical			
Surgical	1090 (37.7)	1083 (36.2)	0.255
non-surgical	1804 (62.3)	1909 (63.8)	
Cancer			
Yes	209 (7.2)	304 (10.2)	<0.001
No	2685 (92.8)	2688 (89.8)	
Department			
Orthopedics	278 (9.6)	280 (9.4)	0.78
Surgery	420 (14.5)	370 (12.4)	0.018*
Internal medicine	983 (34.0)	995 (33.3)	0.582
Neurosurgery	154 (5.3)	175 (5.8)	0.41
Gynecology	555 (19.2)	644 (21.5)	0.028*
Cardiology	183 (6.3)	156 (5.2)	0.077
Urology	157 (5.4)	198 (6.6)	0.062
Other	164 (5.7)	174 (5.8)	0.85

*: p<0.05

### Number of DVT diagnoses and diagnosis rates

Among 2,894 patients in the preoperative group, there were two patients with DVT, and the diagnosis rate was 0.06%. Of the 2,992 patients in the postoperative group, there were 17 patients with DVT, and the diagnosis rate was 0.56%. DVT diagnosis rates were significantly higher in the postoperative group (p=0.0017) (**Table 5**). The number of DVT screenings by lower extremity venous ultrasonography increased from 35 in the preoperative group to 65 in the postoperative group. The DVT diagnosis rate increased significantly in the postoperative group, from 5.7% in the preoperative group to 26% in the postoperative group (p=0.0152) (**Table 6**).

**Table 5:** Table 5 The outcome of DVT and PTE before/after execution of DVT prevention protocol in our hospital

Outcomes	Before Protocol	After Protocol	P value
Number of DVT diagnosis (Diagnosis rate)	0.06% (2/2894)	0.56% (17/2992)	0.0017*
Number of PTE diagnosis (Diagnosis rate)	0.03% (1/2894)	0.07% (2/2992)	0.98

*: p<0.05

**Table 6:** Table 6 Diagnostic rate by ultrasonography before/after implementation of DVT prevention protocol in our hospital

Outcomes	Before Protocol	After Protocol	P value
Number of ultrasonography of the lower extremity veins	35	65	—
Diagnostic rate by ultrasonography	5.7% (2/35)	26.2% (17/65)	0.0152*

*: p<0.05

The demographic characteristics of DVT patients diagnosed after implementation of the DVT prevention protocol are shown (**Table 7**). First, as for the patient background, elderly people aged 65 and over accounted for 88% of the total, and the ratio of elderly people aged 75 to 89 was the highest at 76%. In terms of gender difference, the rate of female patients was 71%. Surgical and non-surgical cases were more common than non-surgical cases (surgical 41% vs. non-surgical 59%); 21% of all patients were admitted to the retirement home, a patient population with a high level of care. By departments, orthopedics and internal medicine accounted for 70% of the total. A history of cancer was found in 3 cases. Wells’ score for DVT was ≥2 points, evaluated on admission, in 4 cases, 0 to 1 point in 13 cases. The next section shows the DVT and the situation at the time of diagnosis. There were central type DVT (53%) and peripheral type DVT (47%). Fifty-nine percent of the patients had symptomatic DVTs, such as leg swelling and redness, and 41% had asymptomatic DVT. D-dimer was elevated (>1.0 µg/mL) at the time of diagnosis in 16 patients, except for one patient who did not take a blood test, and the average was 35.4±34.1 µg/mL. In the risk assessment of DVT based on the Venous Thromboembolism Risk Scale,^7)^ 4 patients were at medium risk, 2 at high risk, 2 at highest risk, and 5 patients were not assessed. The four DVT cases diagnosed on the first day of hospitalization were excluded because they represented the highest risk at that time and would affect the interpretation of the results. The DVT prophylaxis measures implemented were: 4 cases were no measures, elastic stockings used alone in 9 cases, elastic stockings combined with intermittent pneumatic compression devices in 4 cases, and no anticoagulation therapy was used. There were four DVT cases diagnosed on the first day of hospitalization with a Wells’ score for DVT of more than 2 points. The other 13 patients were diagnosed with DVT between 5 and 31 days after admission. The other 13 patients were diagnosed with DVT between 5 and 31 days after admission. Signs of each case included clinical signs of DVT (such as swelling and pain in the lower extremities) in 6 cases, an elevated D-dimer (17.8 µg/mL to 48.4 µg/mL) in 4 cases, a diagnosis of DVT after PTE diagnosis in 2 cases, and one case that was discovered at the time of the upper and lower abdominal CT scan it was.

Of the two patients who developed PTE, one had clinical symptoms of PTE.

**Table 7:** Table 7 The demographic characteristics of DVT patients diagnosed after implementation of the DVT prevention protocol

Patient Demographic	n=17	
Age		
16–64	1	6%
65–74	1	6%
75–89	13	76%
≧90	2	12%
Gender		
Male	5	29%
Female	12	71%
Surgical/non-surgical		
Surgical	7	41%
non-surgical	10	59%
Admission from retirement home		
Yes	4	24%
No	13	76%
Department		
Orthopedics	6	35%
Surgery	1	6%
Internal medicine	6	35%
Neurosurgery	3	18%
Urology	1	6%
Cancer		
Yes	3	18%
No	14	82%
Wells’ score for DVT		
≧2	4	24%
≦1	13	76%
Type of DVT		
Proximal	9	53%
Peripheral	8	47%
Clinical sign of DVT		
symptomatic	10	59%
asymptomatic	7	41%
D-dimer (µg/mL)		
No data	1	6%
≦1.0	0	0%
>1.0	16	94%
Risk determination of DVT^a^		
No risk	0	0%
Low risk	0	0%
Middle risk	4	24%
High risk	2	12%
Highest risk	2	12%
No data	5	29%
Methods of prophylaxis DVT		
No prophylaxis	4	24%
GCS	9	53%
GCS+IPC	4	24%
Anticoagulation	0	0%
Date of diagnosis with DVT (After hospitalization)		
≦1 day	4	24%
≧2 days	13	76%
With PTE		
Yes	2	12%
No	15	88%
Clinical sign of PTE^b^		
symptomatic	1	6%
asymptomatic	1	6%

^a^ Deep vein thrombosis was discovered on the day of admission (n=4) were excluded from this analysis. ^b^ Pulmonary thromboembolism was only two cases. Abbreviations: DVT: deep vein thrombosis; PTE: pulmonary thromboembolism; GCS: Graduated compression stocking; IPC: Intermittent pneumatic compression

The demographic characteristics of each case diagnosed with DVT on the day of admission are shown (**Table 8**).

Of the 4 patients, 2 were hospitalized for surgery, and 2 were non-surgery. The type of DVT was 2 proximal type (1 symptomatic and 1 asymptomatic) and 2 peripheral lower extremities localized (2 symptomatic).

Anticoagulation therapy to preventing DVT was not performed in all 4 patients. In one asymptomatic case of proximal DVT, an angiographic CT showed a thrombus in the inferior vena cava.

**Table 8:** Table 8 The demographic characteristics of cases diagnosed with DVT on the day of admission

Patient Demographic	n=4	
Wells’ score for DVT		
≧2	4	100%
≦1	0	0%
Surgical/non-surgical		
Surgical	2	50%
non-surgical	2	50%
Type of DVT		
Proximal	2	50%
Peripheral	2	50%
Clinical sign of DVT		
symptomatic	3	75%
asymptomatic	1	25%
Anticoagulation therapy to prevent DVT		
Yes	0	0%
No	4	100%

Abbreviations: DVT: deep vein thrombosis

### Number of PTE diagnoses and diagnosis rate

Of the 2,894 patients in the preoperative group, there was 1 PTE diagnosis, and the diagnosis rate was 0.03%. Of the 2,992 patients in the postoperative group, there were 2 PTE diagnoses, with a diagnosis rate of 0.07%. There was no statistically significant difference in the rate of PTE diagnosis (p=0.98) (**Table 5**).

The cases of PTE in the postoperative group were diagnosed as DVT after the diagnosis of PTE.

## Discussion

The impetus for establishing the DVT prevention team was an acute PTE that occurred in our hospital during surgery. The patient was injured in a motorcycle fall and was scheduled for open fracture repair of the right upper arm and left lower leg. The patient had severe diabetes mellitus and was scheduled for surgery after blood glucose control, during which he required a 14-day period of bed rest. During the operation, a sudden drop in percutaneous arterial blood oxygen saturation was observed during the change of position, and the operation was interrupted. Subsequently, emergency angiography revealed left and right pulmonary artery thrombosis and the patient underwent catheterized thrombus aspiration and lysis, which saved his life without any special sequelae. In our opinion, DVT developed during the preoperative period, triggered by the intraoperative repositioning of the thrombus and leading to PTE development. As a result of our medical safety management department’s analysis, we found it was difficult to treat the patient based on the DVT prevention guidelines to identify the risk of developing DVT, preventive measures, and early detection.^4,5)^ We have begun to work on developing the DVT prevention protocol as a top priority for systematic DVT prevention. The developed DVT prevention protocol was then disseminated in the hospital by our DVT prevention team.

Our protocol was inspired by the “Venous Thromboembolism Risk Assessment Table”^7)^ by Kobayashi and the “3-Step Evaluation Method”^3)^ by Kuroiwa.

In Japan, since approximately 20% of venous thrombosis is reported to occur in the perioperative period and 80% in the non-perioperative period, DVT prophylaxis was included in all patients aged 16 years and older.^8)^ Yamada et al. reported that DVT was identified in approximately 18% of non-surgical hospitalized patients. The DVT prophylaxis recommended in prevention guidelines should be considered for non-surgical hospitalized patients.^9)^ Many DVTs were found in non-surgical cases in our study, and we believe that our protocol that included not only surgical cases but also non-surgical cases contributed significantly to the improved DVT diagnosis rate in the postoperative group. About 70% of DVT are asymptomatic, and it is difficult to diagnose DVT only based on clinical symptoms. Therefore, screening by ultrasound sonography is necessary for the accurate diagnosis of DVT, including asymptomatic DVT. Still, it is inefficient in terms of time, labor, and economics to perform these procedures in all cases.^10)^ On the other hand, the Wells’ score for DVT is a tool for promoting the diagnosis and treatment of patients suspected of having DVT, based on the patient’s risk factors and clinical symptoms.^11–13)^ The Japanese guidelines suggest that the Wells’ score for DVT is useful as a basic approach to the diagnosis of DVT.^5)^

However, its usefulness has been shown only in outpatients, and some reports suggest that it is not useful in hospitalized patients.^14)^

We focused on the fact that the Wells’ score for DVT does not require many resources such as time, labor, and money, and we thought it would be meaningful to use the Wells’ score for DVT for hospitalized patients, so we incorporated it into the protocol. Our protocol allows for a Wells’ score on the day of admission, which is similar in timing to the outpatient assessment.

In fact, in four cases of DVT diagnosed after the operation, the Wells’ score of ≧2 on the day of admission and a subsequent D-dimer test showing >1.0, vascular ultrasound test of lower extremity revealed a DVT. Assessment with the Wells’ score on the day of admission appears to be excellent for screening for DVT that has already occurred and DVT that occurred after admission. However, it is impossible to screen for all DVTs, including asymptomatic DVTs, as the Wells’ score items are often underestimated without scoring them. In the future, consideration should be given to improving the accuracy of individual staff members’ evaluations and reviewing the items to be scored on the Wells’ score.

According to our protocol, patients with a Wells’ score ≤1 point on admission have a low probability of DVT. The risk of developing DVT is assessed using the “Venous Thromboembolism Risk Assessment Table” when the patient has been in bed for more than 48 h. Also, our risk assessment method differs from that of Kobayashi in that we assessed the risk of developing DVT using an assessment sheet for non-surgical cases. Then, the risk was stratified from the total score and recommended DVT prevention measures to prevent DVT development.

However, our results show that the incidence of DVT is increasing. Looking at the trend of 13 cases, except for four cases of probable brought-in DVT, the venous thromboembolism risk assessment results did not lead to prophylaxis, even though all of the cases were at intermediate risk or higher, except for the unknown cases. This may be due to inadequate DVT prevention measures (e.g., lack of anticoagulation to prevent DVT development, lack of preventive measures, and mismatch between risk and recommended preventive measures). It is necessary to analyze and validate the protocol and to introduce tools such as the “Venous Thromboembolism Risk Assessment Table (for surgical cases)” and the Caprini score,^15)^ whose risk varies depending on the surgical technique.

DVT prevention measures have been implemented for non-surgical cases postoperatively, but the number of DVT diagnoses is increasing. However, preventive measures have been implemented. This result may be from the use of the protocol and the awareness of staff to screen for DVTs that had not been detected before. The number of lower extremity venous ultrasonography procedures increased 1.8-fold, and the diagnosis rate of DVT increased in the postoperative. Improving the DVT diagnosis rate is an important factor in early treatment and prevention of proximal extermination of thrombosis after DVT onset and the prevention of death from PTE.

Of the two postoperative PTE cases, one occurred on the 13th day of hospitalization in a patient with multiple strokes, and the other occurred on the first day after laparoscopic lymph node biopsy in a patient with suspected malignant lymphoma. Both patients had a Wells’ score for DVT of 0 points on the day of admission, suggesting that DVT prevention measures may have been inadequate. In the future, it is necessary to analyze cases of DVT and to validate the validity of DVT prevention measures and methods for assessing the patient-specific risk of developing DVT, and to continue to take approaches to prevent the development of DVT and PTE.

A review investigating the usefulness of team interventions found that implementing a variety of system-wide strategies for inpatients at risk of venous thromboembolism with multifaceted interventions, including staff education, has the potential to prevent DVT.^16)^ We believe our DVT prevention team’s activities and protocols help improve the DVT diagnosis rate and preventing PTE onset through multifaceted interventions, such as “risk identification and disease recognition,” “prevention,” “early detection and early diagnosis,” “early treatment,” and “hospital system development,” as suggested in the aforementioned recommendations.

## Conclusion

The intervention of the DVT prevention team helped to disseminate the DVT prevention protocol systematically.

## Additional Note

The study was presented at the 38th Annual Scientific Meeting of the Japanese Society of Phlebology (2018, Yokohama, Japan).

## Notes

This is a translation of Jpn J Phlebol 2019; 30(3): 285–293.
